# Update in treatment options for congenital and immune thrombotic thrombocytopenic purpura

**DOI:** 10.3389/fphar.2026.1841673

**Published:** 2026-06-18

**Authors:** George M. Rodgers

**Affiliations:** Division of Hematology and Hematologic Malignancies, Huntsman Cancer Institute, University of Utah Health Sciences Center, Salt Lake City, UT, United States

**Keywords:** ADAMTS13, caplacizumab, plasma exchange, rituximab, thrombotic thrombocytopenic purpura, Upshaw-Schulman syndrome

## Abstract

Thrombotic thrombocytopenic purpura (TTP) is a thrombotic microangiopathy resulting either from congenital deficiency (cTTP) or acquired (immune) deficiency (iTTP) of A Disintegrin and Metalloprotease with ThromboSpondin-type 1 motif, member 13 (ADAMTS13). Deficiency of ADAMTS13 leads to disseminated platelet thrombosis and organ dysfunction. High mortality of cTTP is prevented by plasma infusion to replace the deficient protease, or more recently by infusion of recombinant ADAMTS13. Standard treatment of iTTP includes steroids, plasma exchange, and rituximab, with or without caplacizumab. Although standard treatment of iTTP improves mortality, refractory cases persist, indicating the need for additional treatment options. This review summarizes the status of novel treatment options for cTTP and iTTP, including additional recombinant ADAMTS13 products, ADAMTS13 gene therapies, plasma cell-directed therapies (bortezomib, daratumumab) as well as novel inhibitors of von Willebrand factor activity.

## Introduction

**FIGURE 1:**
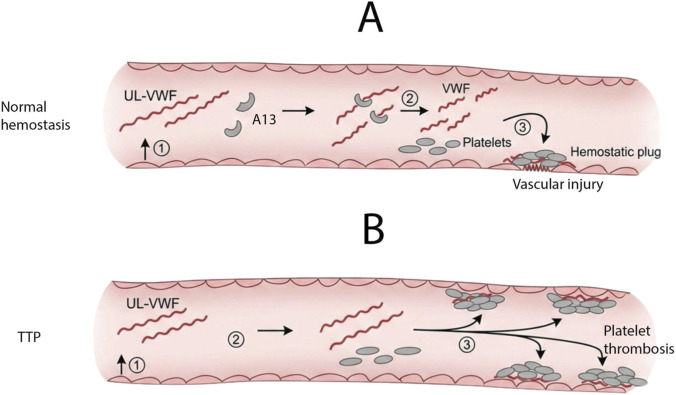
Normal hemostasis and pathologic hemostasis of TTP. **(A)** Depicts normal hemostasis (1) Vascular endothelium secretes von Willebrand factor (VWF) in the form of ultra-large polymers (multimers) which are appropriately processed by proteolytic cleavage of ADAMTS13 (A13). (2) Normal-sized VWF multimers circulate with platelets and only interact with each other and the vessel wall in the event of vascular injury (3), to form the initial hemostatic plug (primary hemostasis) **(B)** depicts aberrant hemostasis associated with TTP. Ultra-large VWF multimers are secreted, but due to ADAMTS13 deficiency (either from congenital absence or auto-antibodies to ADAMTS13) (1), the UL-VWF multimers are not processed and (2) inappropriately induce disseminated platelet thrombosis, resulting in the clinical disorder, TTP.

The thrombotic microangiopathies (TMA) are a heterogeneous group of disorders that have in common hemolytic anemia, consumptive thrombocytopenia, and organ dysfunction (Zhou et al., 2025). Thrombotic thrombocytopenic purpura (TTP) is a unique TMA in that both congenital (cTTP) and acquired (immune) (iTTP) forms of the disorder exist. Both result from deficiency of ADAMTS13 (A disintegrin and metalloprotease with thrombospondin-type 1 motif, member 13), a key enzyme that regulates von Willebrand (vWF) activity. vWF is synthesized by vascular endothelium and megakaryocytes; ADAMTS13 processes vWF by proteolytic cleavage of ultra-high molecular weight polymers (multimers) of vWF to normal vWF that circulates in plasma to mediate platelet adhesion (Sukumar et al., 2021) (Figure 1). Deficiency of ADAMTS13 results in lack of appropriate cleavage of vWF, inappropriate platelet thrombosis, and organ dysfunction (Figure1). cTTP is due to mutations in the ADAMTS13 gene (Markham-Lee et al., 2023), while iTTP results from auto-antibodies to ADAMTS13 (Tsai and Lian, 1998; Furlan et al., 1998).

**FIGURE 2:**
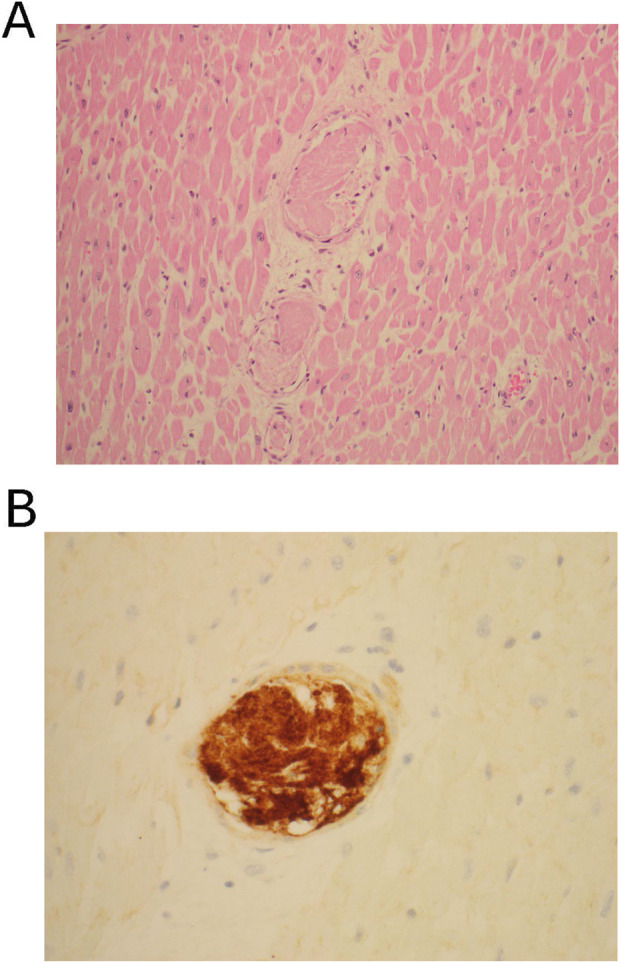
Histopathology of iTTP. Tissue samples are from a fatal case of iTTP. **(A)** Depicts hyaline thrombosis in myocardium (arrow). **(B)** Depicts tissue stained with anti-CD61 (an antibody to the platelet membrane glycoprotein IIb IIIa receptor) indicating that the hyaline thrombosis is composed of platelets.

Figure 2 depicts the classic pathologic feature of TTP-hyaline thrombosis of terminal arterioles and capillaries (Figure 2A). The thrombus consists of platelets as indicated by the immunostained image (Figure 2B).

cTTP has been routinely treated by fresh-frozen plasma infusion to replace the deficient protease (Zheng et al., 2020). iTTP has been routinely treated with plasma exchange (PLEX) and immunosuppression with steroids and rituximab, with or without caplacizumab, an inhibitor of vWF activity (Zheng et al., 2020). This review summarizes the current treatment approach for both cTTP and iTTP and provides an update on investigational therapies for both disorders.

## Methods

A narrative review was conducted to analyze current and potential new treatments for iTTP and cTTP. The PubMed database was searched for articles from 1 January 2010 through 1 May 2026 that included the following keywords “thrombotic thrombocytopenic purpura, Upshaw-Schulman syndrome, ADAMTS13, plasma exchange, rituximab, and caplacizumab.”

This review focused on pivotal TTP clinical trials, including HERCULES and TITAN together with systematic reviews and meta-analysis. The review also included prior foundational reports and more recent discoveries, as well as cost-related information to provide clinicians with relevant information on current treatment options for TTP.

### cTTP

cTTP is an autosomal recessive disorder with an estimated prevalence of 0.5–2 cases per million population worldwide (Asmis et al., 2022). The disorder is also known as Upshaw-Schulman syndrome described in 1960 (Schulman et al., 1960) and 1978 (Upshaw, 1978) in separate case reports. The pediatric patients had a TMA responsive to plasma infusion. In subsequent years, fresh-frozen plasma (FFP) infusion became the standard of care for cTTP, with patients receiving 5–10 ml/kg FFP biweekly for prophylaxis, and at least 10 ml/kg dose used to treat acute cTTP episodes. The 10 ml/kg dose increases plasma ADAMTS13 levels >10% which is usually sufficient to prevent disease activity (Fujimura et al., 2015). Because of variable patient pharmacokinetics, monitoring of ADAMTS13 levels is recommended to optimize FFP efficiency. Certain acute episodes of cTTP may require daily FFP infusions or plasma exchange (Coskun et al., 2025).

The genetic basis for cTTP was reported 25 years ago (Levy et al., 2001); mutations in the ADAMTS13 gene were confirmed. All domains of the ADAMTS13 protease have been associated with mutations linked to disease activity, with over 200 gene mutations described to date (Stubbs et al., 2025). Recombinant ADAMTS13 (apadamtase alfa, rADAMTS13) was approved by the United States Food and Drug Administration for treatment of cTTP in 2023. The pivotal clinical trial comparing FFP vs. rADAMTS13 was reported in 2024 (Scully et al., 2024). Table 1 summarizes treatment options for cTTP.

**TABLE 1:** Treatment options for cTTP.

Recommended treatments
rADAMTS13 (preferred)
FFP (if rADAMTS13 is not available) Dosing: acute episodes- 10–15 ml/kg/day for several days Prophylaxis – 10–15 ml/kg every 1–3 weeks
**Not Recommended**
Intermediate-purity VWF/FVIII Concentrates
Whole blood
Cryoprecipitate
Cryosupernatant

Recommendations are from Zheng et al. (2025).

Abbreviations: cTTP, congenital thrombotic thrombocytopenic purpura; rADAMTS13, recombinant A disintegrin and metalloprotease with thrombospondin type 1 motif, member 13; VWF, von Willebrand Factor; FVIII, factor VIII.

In the pivotal study of 48 patients, there were no acute TTP events in the rADAMTS13 cohort, while the FFP group experienced one such event (Scully et al., 2024). Treatment-related adverse events in the rADAMTS13 group occurred at a 9% rate vs. 48% in the FFP group. No patient in the rADAMTS13 group had adverse events leading to drug interruption or discontinuation; however, 8 patients in the FFP group had such events (Scully et al., 2024). The favorable results of safety and efficacy of rADAMTS13 in this trial led the International Society on Thrombosis and Haemostasis to recommend rADAMTS13 over FFP to treat cTTP in its most recent updated treatment guidelines (Zheng et al., 2025). The recommended dose for prophylaxis of cTTP using rADAMTS13 is 40 units/kg IV every other week (Zheng et al., 2025).

Alternative treatment options for cTTP include intermediate-purity factor VIII–vWF concentrates that also contain ADAMTS13 activity, as well as other blood bank products such as whole blood, cryoprecipitate, and cryosupernatant (Alwan et al., 2019). These alternative products have disadvantages such as transfusion reactions, potential pathogen transmission, and variable efficacy; the preferred cTTP treatment at this time is rADAMTS13, or FFP infusions if rADAMTS13 is not available (Zheng et al., 2025). There is a potential for immunogenicity with rADAMTS13 use, but neutralizing antibodies were not reported in clinical trials.

### Novel treatment approaches for cTTP

A variety of targeted treatment options for cTTP are under investigation, although none to date have led to trials in cTTP patients (Moroniti et al., 2024). These include loading ADAMTS13 into platelets followed by infusion into patients, infusing mRNA-encoded ADAMTS13 into patients, and gene therapy with ADAMTS13. Platelet-delivered ADAMTS13 appears to have the most preclinical data (Moroniti et al., 2024).

### iTTP

Annual incidence estimates are 2–6 cases per million, and prevalence estimates are 13 cases per million, with women affected more than men (Pishko et al., 2025). Associated conditions with iTTP include auto-immune disorders, human immunodeficiency virus infection, and pregnancy (Pishko et al., 2025). Patients with iTTP will typically have ADAMTS13 activity levels <10% of normal with antibodies (inhibitors) to ADAMTS13 being present.

Identifying an inhibitor to ADAMTS13 is important in confirming a diagnosis of iTTP and excluding cTTP. Some iTTP patients will have negative inhibitor test results associated with antibodies that increase ADAMTS13 clearance; these antibodies may not be detected by standard assays. One study found that in a cohort of iTTP patients with negative initial inhibitor results, 21% of this group subsequently were found to have detectable anti-ADAMTS13 antibodies (Simon et al., 2025).

Plasma exchange (PLEX) with high-dose steroids has long been considered standard therapy for iTTP, with the exchange procedure removing antibodies to ADAMTS13 and plasma infusion replacing the deficient protease (Zheng et al., 2020) (Table 2).

**TABLE 2:** Treatment options for iTTP.

Recommended treatments
PLEX + Steroids (strong recommendation) (prednisone 1 mg/kg/day orally or methylprednisolone 1000 mg IV daily for 3 days)
Rituximab (conditional recommendation)
Caplacizumab (conditional recommendation)

Recommendations are from Zheng et al. (2025). Steroid dose recommendations are from Pishko et al. (2025).

Abbreviations: iTTP, immune thrombotic thrombocytopenic purpura; PLEX, plasma exchange.

The 2020 and 2025 ISTH guideline recommendations for iTTP treatment give “strong” recommendations for using PLEX and steroids, but “conditional” recommendations for rituximab and caplacizumab (Zheng et al., 2020; Zheng et al., 2025) (Table 2). A “strong recommendation” has characteristics of being supported by credible research that makes the need for additional research unlikely, and most clinicians would follow the recommendation. A “conditional recommendation” has characteristics of being supported by most clinicians and patients, but not all, and additional future studies will likely strengthen the recommendation (Zheng et al., 2025).

Over this 5-year period, there has been an accumulating large body of evidence favoring the use of both of these latter therapies, but there have not been randomized, controlled trials with convincing data to permit rituximab and caplacizumab to achieve a “strong” recommendation. The only randomized clinical trials in iTTP have been the TITAN and HERCULES trials of caplacizumab (discussed below). Table 3 summarizes a timeline of iTTP treatment development.

**TABLE 3:** Timeline of iTTP treatment development.

1991	PLEX superior to plasma infusion	Rock et al. (1991)
1991	Steroid monotherapy effective in 28% of iTTP patients	Bell et al. (1991)
2003	Rituximab efficacy-case report	Zheng XL et al. (2003)
2011	Rituximab phase 2 trial with PLEX	Scully et al. (2011)
2016	Caplacizumab TITAN trial	Peyvandi et al. (2016)
2019	Caplacizumab HERCULES trial	Scully et al. (2019)
2019	FDA approval for caplacizumab	​
2022–2025	Plasma cell-directed therapies for iTTP-case reports	Chen and Shortt (2022) Weisinger et al. (2025)
2023	FDA approval for rADAMTS 13	​
2024	rADAMTS13 effective in refractory iTTP-case report	Bendapudi et al. (2024)
2026	rADAMTS13 effective in pregnancy iTTP-case report	Cauchois et al. (2026)

Abbreviations: PLEX, plasma exchange; iTTP, immune thrombotic thrombocytopenic purpura; rADAMTS13 recombinant.

The efficacy of rituximab in treating iTTP was first reported by Zheng et al. (2003) and later evaluated in systematic review and meta-analysis including 9 cohort studies comparing outcomes of iTTP patients treated with rituximab vs. conventional therapy. The relapse rate was 16% in the rituximab group vs. 33% in the control group (p = 0.02); the mortality rate was 3% in the rituximab group vs. 11% in the control group (p = 0.03) (Owattanapanich et al., 2019).

Limitations to this meta-analysis include the lack of randomized, controlled trials with rituximab, possible patient selection bias, and possible publication bias. Despite these limitations, the United States Thrombotic Microangiopathy Consortium iTTP Registry characterizes rituximab use as “increased steadily over time” (Chaturvedi et al., 2022) because studies demonstrate decreased rates of iTTP relapse and improved mortality (Chaturvedi et al., 2022; van de Louw et al., 2021).

European use of rituximab upfront in iTTP therapy is very common; Coppo et al. reported that in their iTTP patient study, all patients received early rituximab (Coppo et al., 2021). The typical dose regimen for rituximab in iTTP is 375 mg/m2 IV infusion weekly for 4 weeks.

Caplacizumab is the newest drug approved for iTTP therapy; it is a nanobody that targets the A1 domain of vWF, inhibiting interaction of vWF with platelets (Duggan, 2018). This interrupts platelet thrombosis which is the pathophysiologic basis for TTP. Caplacizumab has a therapeutic advantage over other iTTP treatments because it immediately inhibits platelet thrombosis, while PLEX and immunosuppression will take days to weeks to achieve this outcome, and for some patients, PLEX and immunosuppression will be ineffective. Pishko et al. (2025) have summarized the clinical trials of immunosuppression therapy and caplacizumab.

Two randomized, controlled trials demonstrated the clinical benefit of caplacizumab in iTTP treatment. The TITAN study published in 2016 (Peyvandi et al., 2016) reported that caplacizumab-treated patients had more rapid platelet count normalization than the placebo group (p = 0.005), fewer iTTP exacerbations (3 vs. 11), and fewer patient deaths (zero vs. 2). The latter two trial endpoints did not achieve statistical significance, but the trends favored caplacizumab. There were more bleeding adverse events in the caplacizumab group (Peyvandi et al., 2016).

The subsequent larger HERCULES trial was published in 2019 (Scully et al., 2019) and confirmed the clinical benefit of caplacizumab. The time to normalization of the platelet count was significantly shorter in the caplacizumab group. A prespecified composite endpoint of TTP-related death, TTP recurrence, or a major thromboembolic event occurred in 9 caplacizumab-treated patients vs. 36 placebo-treated patients (p < 0.001). Refractory TTP occurred in no caplacizumab-treated patients vs. 3 patients in the control group. The most common adverse event in the trial was mucocutaneous bleeding which occurred in 65% of caplacizumab-treated patients vs. 48% of placebo-treated patients (Scully et al., 2019).

The FDA-recommended dosing of caplacizumab is 11 mg IV immediately, followed by 11 mg SC daily, (following PLEX) until improvement in ADAMTS13 activity levels >10–20%. Daily 11 mg SC injections up to 30 days are recommended (if needed), with an additional 28 days of treatment recommended for persistent disease (Cablivi package insert, 2024). Caplacizumab was recently FDA approved for use in iTTP patients as young as 12 years.

Caplacizumab is a controversial treatment for iTTP because it is an expensive drug, its use is associated with a bleeding risk (acquired von Willebrand disease), and the pivotal clinical trial data did not clearly demonstrate a mortality advantage. On the other hand, caplacizumab use was associated with more rapid platelet count recovery, fewer PLEX sessions, and reduced incidence of exacerbations and refractory disease; all of these events would likely reduce hospital stay length and overall cost. The cost issue and bleeding risk potential of the drug may be minimized by alternate-day dosing use of the drug. Kuhne et al. described an alternate-day dosing regimen of caplacizumab which maintains drug efficacy with cost reductions and potential reduced bleeding risks (Kuhne et al., 2022). Despite these concerns and the lack of a “strong” recommendation for its use in the ISTH guidelines (Zheng et al., 2025), caplacizumab is increasingly used to treat iTTP (discussed below).

### Investigational treatment approaches for iTTP

Unlike the novel treatments for cTTP which for the most part have not started in clinical trials, numerous investigational treatment approaches to iTTP are rapidly emerging (Tables 3, 4). Randomized trials comparing the two treatment approaches have not yet been performed, and caution is necessary in extrapolating these preliminary results to the general iTTP population.

**TABLE 4:** Novel treatment options for iTTP.

Treatment	Comments
Steroids, rituximab, caplacizumab	An emerging option, especially in Europe. PLEX is only done for non-responsive patients
Alternative anti-CD20 therapy: Obinutuzumab Ofatumumab	For patients intolerant of or non-responsive to rituximab
rADAMTS13 and its muteins: Wild-type rADAMTS13 GC1126A	Muteins may be less susceptible to inhibition by autoantibodies
Plasma cell-directed therapy: Bortezomib Daratumumab	These drugs may be more effective in inhibition of antibody production for patients non-responsive to anti-CD20 therapy

Abbreviations: iTTP, immune thrombotic thrombocytopenic purpura; PLEX, plasma exchange; rADAMTS13, recombinant ADAMTS13.

### Upfront therapy with steroids, rituximab, and caplacizumab (no PLEX)

The disadvantages of PLEX in iTTP treatment combined with improved patient outcomes with steroids, rituximab and caplacizumab have resulted in trials in which PLEX is omitted from the treatment regimen. This treatment approach is increasingly under study, especially in Europe. A 2024 publication and a presentation at the 2025 ISTH meeting reported promising data with this regimen. The 2024 publication compared outcomes of a cohort of iTTP patients receiving standard treatment with PLEX vs. a cohort receiving steroids, rituximab, and caplacizumab (no PLEX). There were no significant differences in platelet responses, exacerbations, or iTTP-related deaths (Kuhne et al., 2024). A 2025 ISTH abstract summarized results of the MAYARI trial from Europe. (Coppo et al., 2025a); 46 iTTP patients were treated with steroids, rituximab, and caplacizumab (no PLEX). Remission was achieved in 95% of patients and 5% of patients required PLEX (Coppo et al., 2025a). However, this trial excluded patients with severe neurologic or cardiac disease. This promising upfront therapy approach remains investigational and requires additional studies including iTTP patients with severe disease.

Two recent reports support early use of caplacizumab in treatment of iTTP. The Capla 1,000+ project compared 1,015 iTTP patients treated with PLEX, steroids +/− rituximab and caplacizumab vs. a historic control group of 510 patients treated with PLEX and steroids +/− rituximab. Survival was statistically significantly higher in the caplacizumab group. The caplacizumab group also required fewer PLEX sessions and had fewer exacerbations and refractoriness. Major bleeding rate in the caplacizumab group was 2.4% (Coppo et al., 2025b).

A recent meeting abstract (Thrombosis and Hemostasis Summit of North America) summarized a literature review and meta-analysis on the efficacy and safety of caplacizumab in treating iTTP (De Matos et al., 2026). These authors surveyed the literature for studies comparing caplacizumab plus standard of care (SOC)-PLEX, immunosuppression vs. SOC alone. Six studies were identified including 2,054 patients (1,295 receiving caplacizumab plus SOC vs. 759 receiving SOC). Caplacizumab treatment significantly reduced all-cause mortality, refractoriness, and exacerbations. Most bleeding events associated with caplacizumab were mucocutaneous and manageable (De Matos et al., 2026).

### When to escalate therapy?

**FIGURE 3:**
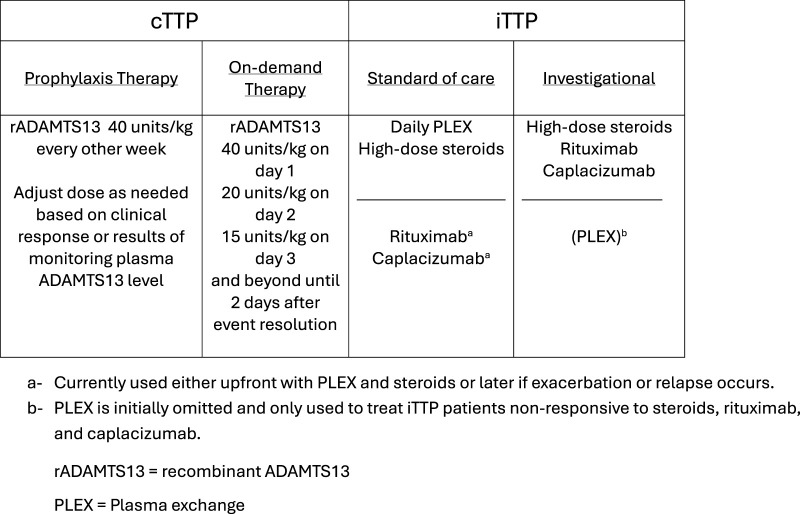
Treatment algorithms for cTTP and iTTP. The cTTP recommendations are taken from the rADAMTS13 product package insert. Two algorithms are shown for iTTP treatment-standard of care (based on guideline recommendations) and an investigational treatment algorithm in which PLEX is omitted. Rituximab is not FDA-approved to treat iTTP, but caplacizumab is approved. Abbreviation: cTTP, congenital thrombotic thrombocytopenic purpura; iTTP, immune thrombotic thrombocytopenic purpura.

Deciding when to escalate therapy depends on which initial therapy has been used to treat iTTP. If standard of care therapy is used (Figure 3), and if the patient has persistent hemolysis (LDH > 1.5 times the upper limit of normal) and platelet count remains <50 × 10^9^/L after 5 sessions of PLEX, treatment escalation should be considered, including adding rituximab +/− caplacizumab (Pishko et al., 2025).

If the investigational arm (Figure 3) is being used to treat iTTP, and there is no improvement in hemolysis parameters and platelets remain <50 × 10^9^/L after 5 days, PLEX should be started.

If patients in either treatment arm persist with refractory disease despite prior therapy with steroids, PLEX, rituximab, and caplacizumab, consideration should be given to using an alternative anti-CD 20 antibody or plasma cell-directed therapy (discussed below) (Weisinger et al., 2025).

### Alternative anti-CD 20 monoclonal antibody therapy

Rituximab use is associated with adverse events, especially infusion-related events, because it is a murine-derived product. Additionally, 10%–15% of rituximab-treated iTTP patients fail to respond in terms of failure to achieve platelet count recovery or failure to eradicate the inhibitory autoantibody to ADAMTS13 (Giannotta et al., 2025). Ofatumumab and obinutuzimab have advantages over rituximab because they are humanized or fully human anti-CD20 monoclonal antibodies with less immunogenicity. Although data is limited at this time, promising results have been reported for ofatumumab and obinutizumab in refractory iTTP.

A United Kingdom registry of 15 patients treated with one or the other antibody therapies reported a 92% complete remission rate (Doyle et al., 2022). Another report of 60 patients with refractory iTTP found an 85% response rate in recovery of ADAMTS13 activity (Weisinger et al., 2025). The subgroup of patient’s refractory to prior rituximab therapy had a 77% response rate (Weisinger et al., 2025). Adverse event rates in both studies were low (Doyle et al., 2022; Weisinger et al., 2025).

### Plasma cell-directed therapy

iTTP patients may experience persistent disease despite B-cell depletion with rituximab or other anti-CD20 therapies, leading to studies investigating plasma-cell directed therapies such as bortezomib (a proteasome inhibitor) or daratumumab (an anti-CD38 monoclonal antibody) in refractory iTTP. In a literature review of case reports and case studies (24 total patients) (Chen and Shortt, 2022) and in a retrospective, observational, multicenter study (Giannotta et al., 2025), response rates to bortezomib were 77% and 59%, respectively. The latter study reported a mild to moderate adverse event rate of 47% (Giannotta et al., 2025).

Fewer refractory iTTP patients have been treated with daratumumab. One study of 2 patients found that daratumumab rapidly achieved a long-lasting remission in both patients (van den Berg et al., 2022). A 2025 review of refractory iTTP management identified a total of 11 published cases of iTTP patients who received daratumumab after rituximab failure; 9 of the 11 patients achieved an ADAMTS13 response (Weisinger et al., 2025). Adverse event rates were low (Weisinger et al., 2025).

### rADAMTS13

To date, 2 case reports describe use of rADAMTS13 in iTTP patients. Bendapudi et al. treated a refractory iTTP patient with rADAMTS13 resulting in rapid improvement (Bendapudi et al., 2024). This patient had a high-titer inhibitor level, but high doses of rADAMTS13 (80 units/kg twice daily) neutralized the inhibitor and restored adequate ADAMTS13 activity. A second case report used rADAMTS13 successfully in a pregnant patient with relapsing iTTP; a single dose of 50 units/kg was used (Cauchois et al., 2026). A phase 2b randomized clinical trial (NCT05714969) is recruiting patients to further evaluate rADAMTS13 in iTTP (Bendapudi et al., 2024).

ADAMTS13 muteins have been proposed as potential novel therapy of iTTP. Jian et al. developed a gain-of-function rADAMTS13 variant that was resistant to iTTP inhibitors (Jian et al., 2012). Whether certain muteins could avoid neutralization of protease activity from a spectrum of iTTP inhibitors without inducing anti-drug antibodies is unknown. Development of ADAMTS13 muteins remains in preclinical investigation.

### N-acetylcysteine (NAC)

NAC reduces disulfide bonds in proteins, including von Willebrand factor; this decreases multimeric size and reduces the potential for platelet thrombosis. A case series of 12 patients with iTTP (2 with refractory iTTP) were treated with NAC (150 mg/kg/day) in addition to PLEX and steroids, with or without rituximab (Espanol et al., 2023). No adverse events were reported, and all patients had complete remissions (Espanol et al., 2023).

### ARC 1779

ARC1779 is a modified DNA/RNA aptamer that targets the A1 domain of vWF, an activity similar to that of calpacizumab (Knobl et al., 2009). However, its use in cTTP and iTTP patients is limited to a few case reports (Knobl et al., 2009; Cataland et al., 2012).

### Anfibatide

Anfibatide is a platelet glycoprotein (GP) 1b antagonist, derived from snake venom; it blocks the GP1b-vWF interaction (Zheng et al., 2016). However, to date only one clinical study has been reported; a phase 1 trial in healthy volunteers that demonstrated its mechanism of action and safety (Li et al., 2021).

## Perspective

### cTTP

rADAMTS13 is the recommended standard of care product to treat cTTP (Figure 3), with FFP being an acceptable option if the recombinant product is not available. Investigational therapies for cTTP are in early development with no likely progression to clinical trials in the near future.

### iTTP

In contrast with cTTP, there is substantial progress in development of new options for treatment of iTTP. Figure 3 summarizes iTTP treatment algorithms using the standard of care approach and the investigational approach. The “cutting edge” investigational paradigm is to initially treat patients with steroids, caplacizumab, and rituximab upfront, without PLEX, reserving PLEX for the minority of patients who fail to respond to initial therapy (Kuhne et al., 2024; Coppo et al., 2025a). Current treatments reduce relapse and mortality of iTTP; there is now an increasing focus on preventing the long-term morbidity and complications of iTTP seen in survivors.

Even after achievement of clinical remission, patients remain at risk of neurocognitive issues, cardiovascular complications, and premature mortality (Pishko et al., 2025). In a 2018 report on 77 TTP survivors in the Oklahoma TTP registry, 21% subsequently died before their expected age of death (George, 2018). Neurocognitive complications of iTTP are being increasingly recognized, including ischemic stroke and depression (Pishko et al., 2025; Selvakumar et al., 2023).

The pathogenesis of these long-term complications likely relates to disseminated platelet thrombosis. In one report, 40% of iTTP patients in remission demonstrated vascular ischemic changes suggestive of silent cerebral infarction on brain MRI (Cataland et al., 2011), and a prospective study reported that 50% of iTTP patients in remission have brain MRI findings consistent with ischemic infarction which correlated with cognitive impairment (Yu et al., 2022).

A recent prospective study followed 22 iTTP survivors over a 1-year period, monitoring brain MRI, CT-perfusion studies, blood-brain barrier (BBB) assessment, and cognitive testing (Hannan et al., 2026). At baseline 82% of patients had abnormal brain MRI, and all patients had compromised BBB integrity. Over 1 year, BBB function improved (but not to normal), serial MRI revealed brain volume loss, and diffusion imaging showed decreased white matter integrity (Hannan et al., 2026). These results indicate that brain injury and cognitive dysfunction occur early in iTTP, and progress even after remission.

These observations suggest that rapid and early prevention of platelet thrombosis in iTTP patients would be beneficial in not only preventing disease mortality, but also long-term morbidity of iTTP. These data support the concept that early use of treatments that rapidly inhibit platelet thrombosis will be preferable to treatments that are less effective and may take longer to achieve efficacy. At this time, there are only two approved iTTP therapies that can achieve this objective-caplacizumab and rADAMTS13. Caplacizumab achieves this by rapidly inhibiting vWF binding to its platelet receptor, while rADAMTS13 infusion neutralizes the inhibitor, and in large doses, replaces the deficient protease to restore normal vWF processing and prevent further platelet thrombosis. Caplacizumab has been approved for use in iTTP, and rADAMTS13 is currently under study for treatment of iTTP (NCT05714969).

While approved for use in iTTP patients for up to 2 months, caplacizumab has been used for up to 6 months in a refractory iTTP patient, normalizing the platelet count while preventing organ damage from platelet thrombosis (Rodgers et al., 2023). Thus, caplacizumab may be viewed as a “rescue therapy” for refractory iTTP patients. rADAMTS13 may also be an effective rescue therapy in iTTP, but its efficacy and safety in iTTP are still under investigation. Table 5 summarizes the benefits and risks of caplacizumab and rADAMTS13 in treating iTTP.

**TABLE 5:** Comparison of caplacizumab and r-ADAMTS13 in treating iTTP.

​	Caplacizumab	rADAMTS13
Mechanism of action	Inhibits platelet thrombosis by preventing VWF binding to platelet GP1b	Neutralizes antibody to ADAMTS13 and replaces deficient protease activity to normally process VWF and prevent platelet thrombosis
Risks	Bleeding risk (induces acquired VWD) 61% (caplacizumab) vs. 45% (placebo) in the TITAN and HERCULES trials	9% adverse events in the pivotal clinical trial No bleeding risks
Costs	$318,867^*^ (for 11 mg daily for 31 days)	$4.06/unit^*^ (for a 70 kg patient receiving 80 u/kg BID)^†^ 1 week = $318,304 2 weeks = $636,608
Comments	In the 2 trials, bleeding events were mild/moderate Bleeding risk and cost may be ameliorated by alternate-day dosing	​

Abbreviations: iTTP, immune thrombotic thrombocytopenia purpura; VWF, von Willebrand Factor; n VWD, von Willebrand Disease.

^*^ Average wholesale cost to the University of Utah Pharmacy in $USD as of January 2026.

^†^ The 80 u/kg BID dosing of rADAMTS13 is based on Bendapudi et al. (2024). Clinical trials have not reported optimal dosing and therapy duration of rADAMTS13 in iTTP.

Refractory iTTP is defined by a platelet count less than 50 × 10^9^/L with persistent hemolysis (LDH level is >1.5 times the upper limit of normal) after the patient has received at least 5 PLEX sessions (Pishko et al., 2025). The basis of refractory iTTP is persistent anti-ADAMTS13 autoantibody production that is non-responsive to standard immunosuppression. IgG subclass also affects therapy response. During the initial iTTP episode, IgG1 predominates; however, IgG4 dominates at the time of relapse (Sinkovits et al., 2018). IgG4 antibodies are more potent and may evade certain immune clearance mechanisms. Additionally, long-lived plasma cells which resist rituximab immunosuppression persist in secreting ADAMTS13 autoantibodies (Chen and Shortt, 2022).

As shown in Table 5, the cost of 31 days use of caplacizumab in treating iTTP is comparable to 7 days use of high-dose rADAMTS13. The optimal dosing and treatment duration of rADAMTS13 therapy in iTTP has not been determined.

## Summary

It is now just over 100 years ago that Dr. Eli Moschcowitz reported the first recognized case of TTP. The patient was a 16-year-old girl who either had cTTP or iTTP and who developed progressive neurologic impairment and died (Moschcowitz, 1925). In the paper’s discussion, Dr. Moschcowitz concluded that death “resulted from some powerful poison that had both agglutinative and hemolytic properties” (Moschcowitz, 1925). We now understand that the powerful poison suggested by Dr. Moschcowitz was UL-VWF multimers, which in the absence of ADAMTS13 processing result in widespread platelet thrombosis, thrombocytopenia, hemolysis, and organ dysfunction. We now have effective therapies for both cTTP and iTTP as summarized below.

### cTTP

The guideline recommendations for standard cTTP treatment are widely accepted; rADAMTS13 is preferred, if available, and FFP is an acceptable option. Other treatment options for cTTP are unlikely to be available in the foreseeable future.

### iTTP

In contrast, the guideline recommendations for iTTP are controversial, with increasing investigational use of upfront caplacizumab, steroids, and rituximab, omitting PLEX. It is uncertain whether there will ever be randomized trials comparing PLEX, steroids, and rituximab (+/− caplacizumab) vs. caplacizumab, steroids, and rituximab.

Although iTTP outcomes continue to improve, increasing patient and clinician appreciation of long-term complications of TTP will likely lead to expanded early use of caplacizumab or other treatments that can rapidly inhibit platelet thrombosis. One potential competitor to caplacizumab is rADAMTS13 which has the advantage of not increasing bleeding risk. However, if clinical trials of rADAMTS13 in iTTP indicate that very large doses of the drug are routinely necessary to induce remission, using rADAMTS13 in iTTP may be cost-prohibitive. For example, the case report by Bendapudi et al. used 80 u/kg rADAMTS13 twice daily for a total of 53 doses. At the price listed in Table 5, the rADAMTS13 cost to treat a 70 kg patient for that duration would be over $1,200,000.

iTTP is an expensive and burdensome disease to treat. PLEX is labor-intensive and not widely available. Effective drugs to treat iTTP such as rituximab, caplacizumab, and perhaps rADAMTS13 are expensive. It is reasonable to question whether the newer treatment options (upfront rituximab and caplacizumab) are cost-effective and necessary to treat iTTP.

Emerging evidence suggests that it may indeed be clinically useful to employ these treatments early in patient management. The advantages of early therapy to inhibit platelet thrombosis and prevent long-term neurocognitive morbidity and cardiovascular complications were previously discussed. The meta-analysis of caplacizumab therapy for iTTP found not only improved survival with caplacizumab, but also shorter hospital stays and fewer PLEX sessions (De Matos et al., 2026).

A formal cost-benefit analysis is necessary to support cost-effectiveness of caplacizumab (and up-front rituximab), but the initial data on improvements in patient survival and other outcomes supports early use of these drugs to treat iTTP. One cost-effective strategy may be to use routine upfront treatment with caplacizumab (with steroids and rituximab), and if patients develop bleeding, to then substitute rADAMTS13 for caplacizumab. If clinical trials with rADAMTS13 demonstrate that lower doses and shorter treatment duration are effective in iTTP, routine early use of rADAMTS13 may become standard of care.
